# Early Life Nutrition and the Role of Complementary Feeding on Later Adherence to the Mediterranean Diet in Children up to 3 Years of Age

**DOI:** 10.3390/nu14081664

**Published:** 2022-04-16

**Authors:** María Gómez-Martín, David Herrero-Morín, Silvia Arboleya, Miguel Gueimonde, Sonia González

**Affiliations:** 1 Department of Functional Biology, University of Oviedo, 33006 Oviedo, Spain; gomezmarmaria@uniovi.es; 2 Diet, Microbiota and Health Group, Instituto de Investigación Sanitaria del Principado de Asturias (ISPA), 33011 Oviedo, Spain; silvia.arboleya@ipla.csic.es; 3 Pediatrics Service, Centro Atención Primaria Infiesto, SESPA, 33530 Infiesto, Spain; herrerojose@uniovi.es; 4 Department of Microbiology and Biochemistry of Dairy Products, Instituto de Productos Lácteos de Asturias (IPLA-CSIC), 33300 Villaviciosa, Spain

**Keywords:** infant diet, Mediterranean diet, weaning, complementary feeding, breastfeeding

## Abstract

The first years of life represent a window of opportunity to establish proper dietary patterns and to maintain them over time. Our aim was to describe the diet of a cohort of Spanish children, from 2 to 36 months, and to identify the components that could influence the quality of the diet at 24 and 36 months of age. This was a longitudinal prospective study analyzing information from administered questionnaires about general characteristics and food frequency consumption in 97 full-term babies. At 2–3 months of age, only 53.6% of infants were observed to be breastfed. The intake of animal foodstuffs from 12 to 36 months was higher than national recommendations, and the contrary was true for fruits and vegetables. The intake of vitamin D was below European Food Safety Authority recommendations. Moreover, energy intake at 6 months was inversely associated with Mediterranean Diet Score (MDS) at 24 months, whereas vegetables intake was positively associated with MDS at 36 months. These results could be useful in the creation of future guidelines focused on the promotion of breastfeeding and healthy early-life food habits.

## 1. Introduction

The period from conception to the age of two years has been described as a window of opportunity to promote long-term effects contributing to health [1]. Following a high-quality diet starting from early life, characterized by a wide variety of nutritious foods and a balanced intake of macro- and micronutrients for energy, is essential to lower the later risk of diet-related non-communicable diseases [2,3]. Until the age of 6 months, breastfeeding provides all the nutritional requirements for a newborn [4]. However, from this age onwards, there is controversy about the ideal pattern for the introduction of complementary feeding. At this stage, children should begin to be introduced progressively to safe and adequate foods until they reach a diet similar to that of an adult, at approximately two years of age [4]. With independence of the nutritional composition, the consumption of certain food groups, such as fruits and vegetables, provides a wide repertoire of flavors, aromas and textures that will shape the future dietary preferences of the infant towards a more varied and nutrient-dense diet [5]. The Avon Longitudinal Study of Parents and Children (ALSPAC) calculated a complementary feeding utility index based on recommendations [6]. Higher index scores were linked to longer breastfeeding and higher intake of fruits and vegetables and a lower presence of ready-prepared baby foods. In terms of health, higher scores were positively related to intelligence quotient and ‘healthy’ dietary patterns in childhood [6]. In accordance with this, some longitudinal studies demonstrated that a greater proportion of total energy from fruits and vegetables in children was associated with a reduction in the risk of cardiovascular mortality in adulthood, contrary to what occurs for meat, dairy products and sugar supply [7]. Although most previous works in the literature focused on the importance of isolated food groups or particular nutrients, this may hide the fact that different foods are combined as part of the diet, and this implies a high degree of correlation among them. Despite the fact that at present, no validated index has been found to evaluate the quality of diet in children, one of the most recognized beneficial dietary patterns worldwide is the Mediterranean Diet (MD) [8]. Adherence to this pattern, represented by a high content of fruits and vegetables, a low proportion of meat and dairy products and the use of olive oil as culinary lipids [9], from an early age could bring numerous benefits [10,11]. A study that investigated the association between parents’ lifestyle determinants and children’s dietary habits and physical activity levels showed that higher levels of maternal educational and physical activity were positively associated with children’s MD [12].

Based on the above evidence, it seems reasonable to hypothesize that the choice of breastfeeding during the first months of life and, subsequently, a healthy dietary pattern with a high repertoire of foods with high nutritional density, may condition the achievement of a healthier diet later in life. For all these reasons, research on the eating habits of children in this age range is of great relevance for the establishment of health promotion strategies in the early years of life that will improve later health [13]. In this setting, the aim of this study was to assess the diet of a cohort of Spanish children and to identify the components of weaning and complementary feeding that could influence the dietary quality at 24 and 36 months of age.

## 2. Materials and Methods

### 2.1. Sample Recruitment and Study Design

**Figure 1:**
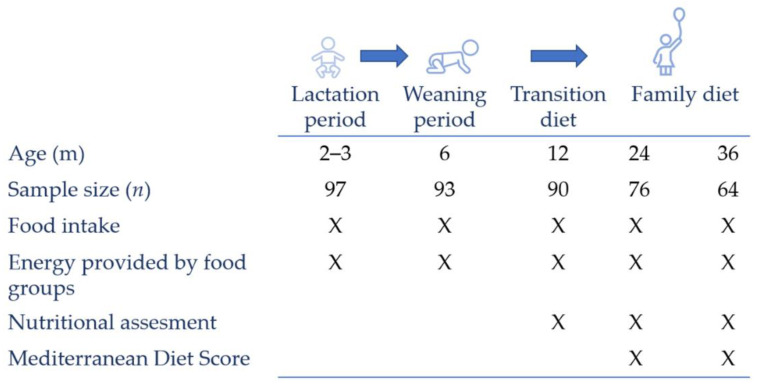
Study cohort and sampling points.X, data available.

The cohort was composed of 97 full-term babies (37–40 gestational weeks) at the lactation period (2–3 months of life), 93 at the weaning period (6 months) and 90 subjects at the transition diet (12 months); meanwhile, the family diet (24 and 36 months) was composed of 76 and 64 subjects, respectively (Figure 1). Participants were recruited through the Primary Care Pediatrics Service in Asturias, on the north coast of Spain, at the first medical consultation. When the legal tutors or caregivers of all participants were informed of the objectives of the study, they signed their written consent. The Regional Ethics Committee of Clinical Research of Asturias (Reference no. 12/16, 03/02/2016) and the Committee on Bioethics of CSIC (Reference no. PCIN-2015-233) evaluated and approved the study procedures. All protocols were performed in line with the principles stated in the Declaration of Helsinki, the Bioethics Convention of University of Oviedo, the Council of Europe’s Convention on Human Rights and Biomedicine and in Spanish legislation on bioethics. The Directive 95/46/EC of the European Parliament and the Council of 24 October 1995 on the protection of individuals regarding the processing of personal data and on the free movement of such data was strictly followed. 

### 2.2. General Characteristics

At baseline, data were collected on the characteristics of the infants (i.e., sex and age) and the type of delivery. In addition, several characteristics related to dietary habits, such as type of breastfeeding, consumption of vitamin and mineral supplements, diet texture or prescription of a therapeutic diet, were assessed at the different sampling times. The height (cm) and weight (kg) of the children to the nearest 0.1 cm and 0.1 kg, respectively, were measured with calibrated and suitable equipment by pediatric nurses. 

Dietary questionnaires were collected at the time of recruitment and at 2–3, 6, 12, 24 and 36 months (Figure 1).

### 2.3. Nutritional Assessment

The children’s diets were registered using a self-administered food propensity questionnaire for the previous week based on the Pilot Study for the Assessment of Nutrient Intake and Food Consumption among Children in Europe (PANCAKE) [14] and adapted for Spanish children and local culinary customs and recipes. Furthermore, food dairies were administered using an online tool. Foods were categorized into 12 food groups according to the European Prospective Investigation into Cancer (EPIC) criteria [15], incorporating another 2 concerning infant products: breast milk and processed infant products. Food groups included: fats (vegetable oils and solid fats); vegetables (bulbs, mushrooms, roots, inflorescences and stem and leaf vegetables); legumes (lentils, chickpeas, soy, beans and peas); fruits (fresh, dried and canned fruits); potatoes and tubers (potato and sweet potato); cereals and cereal products (bread, pasta, flours and grains); meat and meat products; fish (fish and fish products, crustaceans and mollusks); eggs; milk and dairy products (milk, yogurt, dairy dessert, milkshake and fresh, mature and processed cheeses), sweet and desserts (sweets, cake, biscuits, chocolate, honey and others), sweetened beverages (natural and non-natural fruit juices, soft drinks and soy-based beverages) and human breast milk and processed infant products (infant formulas: starter formulas, special starter formulas, follow-up formulas, special follow-up formulas, growing-up milk; infants cereals and infant purees: fruits, fruit and cereals, vegetables, legumes and pasta, meat, fish and others) [16]. 

The questionnaires were completed by the mother, father or caregiver of the child, who received them by e-mail or mobile phone. Specific instructions to complete the questionnaire were detailed at the start of each section and the validated photo album developed by the PANCAKE was used for serving size estimation, taking into account the EU-Menu guidelines [17]. As dietary information was collected over a prolonged period, at different times from birth, an adapted different version of the same questionnaire was used at the lactation period due to the absence of complementary feeding. 

Breastfeeding was assessed in each time period. To calculate the categorical variable, the type of breastfeeding was classified as breastfeeding (including mixed) or infant formula. Regarding the quantitative variable, the volume of breast milk received was estimated by using the mean values reported in the previous studies for each stage of age (780 mL for infants up to 6 months and 600 mL for infants from 7 to 12 months, in the cases of exclusive breastfeeding) [18,19]. For the infant formulas, the volume reported by the parents was used, assuming that the manufacturer’s prescriptions regarding the weight of powdered milk to be dissolved per volume were respected. In mixed-fed infants, based on existing literature, the amount of formula consumed per day was measured, and the remaining volume of formula consumed per day was assumed to be breast milk up to 780 mL from start to 6 months and 600 mL at 12 months [18].

The energy content and nutritional composition was calculated using the food composition tables developed by the Centro de Enseñanza Superior de Nutrición Humana y Dietética (CESNID) [20]. The nutritional composition of maternal breast milk [21], infant formula, cereal products and infant purees was obtained from the processed baby foods composition table [16].

Furthermore, detailed information regarding the type of protein or carbohydrate consumed was extended from the food composition tables published by the United States Department of Agriculture (USDA) [22].

A Mediterranean Diet Score (MDS) was created based on previous studies and adapted [23] for the dietary data obtained at 24 and 36 months of age. 

### 2.4. Statistical Analyses

The results were analyzed using the IBM SPSS software version 25.0 (IBM SPSS, Inc., Chicago, IL, USA). Goodness of fit to the normal distribution was determined by means of the Kolmogorov–Smirnov test. When normality of the variables was not achieved, nonparametric tests were used. In general, categorical variables were summarized as percentages and continuous variables using the medians and interquartile ranges (percentile 25 and percentile 75) or the means and standard deviations for descriptive purposes. The Student’s *t*-test and Kruskal–Wallis test were used to evaluate differences in continuous variables and the Z-test, Fisher’s test and chi-square test for categories variables. Adherence to dietary reference values (DRVs) was calculated using the European Food Safety Authority’s (EFSA) recommendations for children aged 1–3 years [24]. The parameters used were adequate intake (AI) and average requirement (AR). The AI can also be used to determine the proportion of individuals with adequate nutrient intake, while the proportion of the population with usual intakes below the AR provides an estimate of the proportion of the group whose intake does not meet nutrient requirements. In order to analyze the impact of the consumption of the food groups, accounting for 80% of the energy intake at 6 months, on the Mediterranean Diet index score at 24 and 36 months, a stepwise linear regression model was conducted. The adequacy of the major food groups to the national recommendations for this age group was calculated [25]. For this purpose, the lowest portions recommended for vegetables, legumes and milk and dairy products, together with the large size for fish and meat, were established as adequacy criteria. GraphPad Prism 8 and BioRender were used for graphical representations. 

## 3. Results

### 3.1. Description of the Sample and Mediterranean Diet Score Calculation

Mean and median intake of the MDS items at 24 and 36 months by gender is presented in Table 1. Higher consumption (above the median) of fruits, cereals (included potatoes), vegetables, legumes and the ratio of monounsaturated/saturated lipids and lower consumption of meat and dairy products each contributed one point to the total score. Thus, the total MDS ranged from 0 to 7.

The general characteristics of the sample, according to the period studied, are presented in Table 2. As expected, weight and height increased significantly across the follow up (from 5.82 to 15.22 kg and 59.98 to 96.27 cm, respectively). The percentage of breastfeeding decreased from lactation period (53.6%) up to 36 months (7.8%) as well as the percentage of mineral or vitamin supplementation (from 92.7% to 0%). In terms of food texture, there was a significant decrease in the proportion of children fed with puree between 6 and 12 months of age. No significant differences were found for the rest of the variables studied.

### 3.2. Dietary Intake

**Figure 2:**
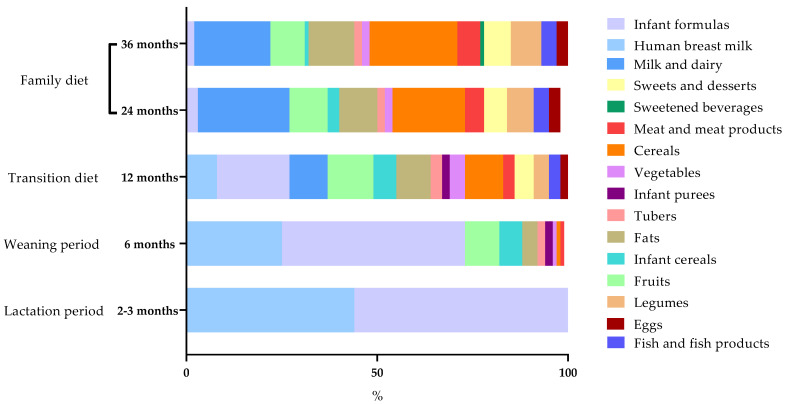
Change in the energy provided by food groups across the follow up.

As expected, during lactation either infant formula or breast milk were the major sources of energy, and at the weaning period, the diet started to become more varied (Figure 2). Fats, vegetables, tubers, fruits and processed baby foods were the first groups included into infants’ diet (Supplementary Materials Table S1). At the so-called transition diet, the contribution of infant formula and breastfeeding to energy intake decreased in favor of dairy products, fruits, infant cereals, fats and tubers, among others. From 12 to 24 months, the principal observed variation derived from the substitution of breast milk and infant formulas with cow’s milk. No differences were found between 24 and 36 months. In order to assess the degree of adherence to the dietary recommendations for children, the daily intake of the major food groups was compared with the recommendations. Intake of protein foods, such as meat and fish, were above recommendations, while fruits and vegetables were below at all ages (Supplementary Materials Figure S1).

### 3.3. Nutritional Status

Infant’s nutritional intakes at 12, 24 and 36 months, including a comparison with the recommended daily values by age, are presented in Table 3. The data revealed an increase in the intake of most nutrients at 24 and 36 months, in respect to 12 months, as expected. For vitamins, the intake of vitamin D in all periods studied; choline and copper at 12 months; vitamin E at 24 and 36 months were under the recommendations. In the case of minerals, the compliance of AI of magnesium increased from 12 to 24 months, but a decreased was observed from 24 to 36 months (Table 3).

**Figure 3:**
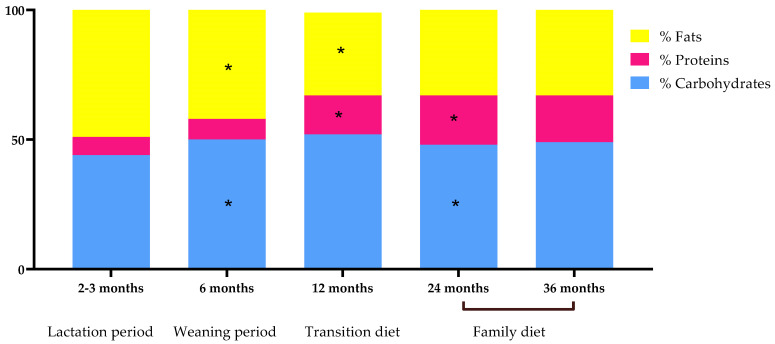
Percentage of the total energy intake provided by each macronutrient across the follow up. Mean daily energy intake: lactation period 538.64 kcal; weaning period 726.74 kcal; transition diet 1046.94 kcal and family diet at 24 months 1147.50 kcal and 36 months 1282.36 kcal. * *p*-Value < 0.05 from Kruskal–Wallis test compared to the previous category.

The variations in the contribution of macronutrient to energy intake during the study is presented in Figure 3. The contribution of protein to energy intake during lactation was 7%, and it increased significantly until the age of 24 months (19%). On the other hand, a decreased was observed in the contribution of fats, from 49% at the lactation period to 33% at 36 months.

### 3.4. Dietary Quality

With the aim of exploring the impact of diet during the weaning period on the adherence to MD at 24 and 36 months, stepwise regression was conducted (Table 4). Energy intake at the weaning period was inversely associated with MDS at 24 months, contrary to vegetables that were positively associated with MDS at 36 months. 

On the other hand, fruits were also negatively associated with MDS at this age (Table 4). It is also noteworthy that a higher percentage of breastfed infants in the lactation period scored better on the MDS at 24 months of age than formula-fed infants. However, these differences were not significant (67.6% vs. 45.0%, *p*-value = 0.051).

## 4. Discussion

Our results increase the existing knowledge about the evolution of diet from the lactation period to the family diet in a Mediterranean cohort, identifying dietary targets that could determine the adherence to a higher quality diet in later age. Considering the importance to health of the first stage of life, the knowledge obtained from these results could be useful in the creation of future guidelines focused on the promotion of healthy habits.

Despite WHO recommendations for exclusive breastfeeding up to 6 months, our results were similar to others with only a 53.6% of the children in the sample breastfed at 3 months [26]. Over time, the duration of breastfeeding was relatively good with 7.8% of the sample being breastfed at 36 months compared to the 2–6% reported by other authors for the interval of ages between 23 and 48 months [27,28]. Therefore, it is still necessary to strengthen strategies to promote it in the first months of life. 

As it has been described, breastfed infants showed a lower weight than formula-fed, 5.63 vs. 6.04 kg at the lactation period [29,30], contrary to what occurs in infant formula-fed babies, who presented a higher weight gain than those breastfed from 2 to 12 months of age [31,32]. This finding could be related with the association between breastfeeding and appetite regulation [33] or by the differences in the amount of energy, macro- and micronutrients and bioactive components between breast milk and infant formulas. In this regard, it is of great interest that children who were breastfed during the lactation period scored better on the MDS at 24 months of age. Even though these results are at the limit of statistical significance, they agree with numerous data about the impact of breastfeeding on a better acceptance of fruits and vegetables later in life [34]. However, considering that at this age infants do not have the capacity to make independent food choices, it cannot be discarded that family dietary habits may be influencing the observed associations. 

The complementary feeding represents a crucial stage in which a balance must be reached in order to guarantee the energetic and nutritional requirements of the child, allowing him/her to have adequate development according to age and considering their limited digestive capacity. In this regard, the European Society for Pediatric Gastroenterology Hepatology and Nutrition (ESPGHAN) defines exclusive breastfeeding for around six months and establishes that the introduction of complementary foods should not occur before 17 weeks but should not be delayed beyond 26 weeks [35].

Apart from infant formula and breast milk, fruits were one of the most consumed foods in the sample at 6 months of age, being the third source of energy followed by infant cereals. This pattern was similar to other Mediterranean countries, such as Italy [36], and different in respect to others, such as England, where baby rice is the starting food [37]. Some food groups considered most allergenic, such as cow’s milk protein (except whole cow’s milk), egg, soy, wheat, peanut, tree nuts, fish and shellfish, were probably introduced into the diet beyond 6 months of age [38,39,40] and, in consequence, their contribution to the total energy intake at this age was inestimable. With independence of food groups, it was observed that energy intake at 6 months was inversely related with MDS at 24 months, which may be in line with other studies showing that the early introduction of high density foods is a predictor of a less healthy diet in the future [41]. Regarding the recommended daily servings by food group, it was observed that there was no agreement at these ages [25,42,43]. When comparing our data with the recommendations of the Spanish Society of Community Nutrition (SENC) [42] for children from 1 to 6 years of age, it was revealed that at 1 year they did not reach the daily recommendations of any food groups, except for red and processed meats, which exceeded the recommendations. On the other hand, at 36 months, a better adherence was observed, with fruits, vegetables and oils being slightly below the recommendations and red and processed meats above them. These discrepancies were due to the different portion sizes for vegetables, fruits and protein groups used to highlight the need for harmonized and standardized portion sizes for this age group [25,42,43].

From 6 to 12 months, a progressive change in food texture was produced from 9.9% of a semi-solid diet to 66.7%, respectively. At 12 months of age, the diet was called a transitional diet because, although all the food groups are consumed, as has been described in previous studies [44], breastfeeding, infant products and a semi-solid consistency in preparations were still maintained. At 24 and 36 months, most of the sample had a regular family diet. Interestingly, at 36 months, a reduction in the intake of vegetables was observed in favor of meat and meat products, which is in line with other studies and representative of the diet of adults in Westernized countries [45,46]. 

At the nutritional level, the percentage of total energy intake provided by each macronutrient was similar to previous studies [47,48,49], showing a moderate increase in protein from 12 to 36 months (15, 19 and 18%, respectively). The requirements for most nutrients were met at all ages studied. Some differences with other studies conducted in the pediatric population were observed. The median energy intake of our children compared to the DONALD study (German population) [50] was higher at all ages; however, compared to the ALSALMA study in Spanish children [48], it was only higher at 12 months. The US study, FITS, showed higher medians at all ages compared to our data [27]. As regards to nutrients, median protein intake at 24 months was higher in our study than in the ALSALMA and FITS (52.2 vs. 46.3 and 50 g/day, respectively) [27,48]. In addition, the median fiber intake in our study was higher for all of the studied times compared with the other studies [27,47,48]. These differences could be due to the presence of several factors including different methodologies, a wider range of age compared, the size of the sample and the different dietary habits among the populations studied.

Regarding micronutrients, vitamin D was the most compromised in the study, in accordance with previous studies [49,51]. The determination of vitamin D intake depends on information from food composition tables and is subjected to seasonal variation. In Spain, children receive vitamin D supplements up to 12 months of age [52] and, as it is a sunny country, the requirements are likely to be covered [51]. However, a previous study in this area of the country suggested that sun exposure may not be enough to cover these deficiencies [53]. Therefore, it would be necessary to clarify whether supplementation with this vitamin for a longer period is needed to cover the nutritional requirements.

Concerning adherence to the Mediterranean Diet at 36 months, it was found that those children who ate vegetables at 6 months of life had better MDS at 36 months. The relationship between the vegetable group and increased adherence to the Mediterranean Diet is well documented [54,55,56]. However, our results also show an inverse association between fruit consumption and MDS, contrary to other works [56,57], the reason for which remains to be elucidated. Among the fruits most consumed in the study sample were banana (52.7%), apple (48.4%), pear (47.3%) and orange (26.9%) followed by plum and strawberry in smaller proportions (4.3% and 2.2%, respectively). 

This study has several limitations related to its observational nature and the collection of dietary information. In interpreting this information, it should be noted that the energy and nutrient contents of processed infant foods were considered, a factor that has hitherto been underestimated in the literature. Regarding the quantification of breast milk energy, it is necessary to mention a limitation of the study. Since it was not possible to record the exact volume of milk produced by the mother, an indirect estimation was made using the mean amounts established in the literature for each age range [18,19]. While the quality of the FFQ depends on the respondent’s memory, its ability to accurately classify energy and all nutrient intakes in children is enhanced by the fact that the questionnaires were adapted from the PANCAKE study and photographs made it easier to interpret. In addition, it allowed for comparison with other studies on the European infant population.

## 5. Conclusions

Due to the low percentage of breastfeeding reported during the lactation period, it would be advisable to establish strategies to promote breastfeeding until at least 6 months of life. The diet of children evaluated at 12, 24 and 36 months was characterized by a low intake of fruits and vegetables and an excess of meat, showing a pattern similar to adults in Western countries. In addition, the results allow us to hypothesize that a lower energy intake and the introduction at 6 months to certain food groups, such as vegetables, are associated with better MDS scores at 24 and 36 months, respectively. Finally, given the large percentage of vitamin D deficiency, it could be suggested as a nutritional target for infants over 12 months of age.

## Figures and Tables

**Table 1:** Mediterranean Diet Score calculation by age group and gender.

	24 Months				36 Months				
	Boys (*n* = 44)		Girls (*n* = 32)		Boys (*n* = 37)		Girls (*n* = 27)		
	Mean ± SD	Median	Mean ± SD	Median	Mean ± SD	Median	Mean ± SD	Median	Score
MUFA/SFA ratio ^a^	1.19 ± 0.35	<1.07 ≥1.08	1.26 ± 0.40	<1.13 ≥1.14	1.23 ± 0.30	<1.19 ≥1.20	1.48 ± 0.45	<1.35 ≥1.36	0 1
Legumes (g/day)	34.85 ± 21.35	<29.93 ≥29.94	27.34 ± 16.13	<24.28 ≥24.29	36.97 ± 17.73	<35.70 ≥35.71	37.50 ± 28.02	<34.28 ≥34.29	0 1
Cereals and potatoes (g/day)	121.07 ± 55.94	<115.34 ≥115.35	105.12 ± 56.91	<98.47 ≥98.48	147.85 ± 52.66	<132.12 ≥132.13	130.24 ± 71.61	<116.42 ≥116.43	0 1
Vegetables (g/day)	101.24 ± 75.13	<85.60 ≥85.61	107.38 ± 98.27	<89.04 ≥89.05	106.83 ± 91.60	<84.85 ≥84.86	114.40 ± 143.73	<64.28 ≥64.29	0 1
Fruits (g/day)	173.95 ± 86.96	<167.23 ≥167.24	186.63 ± 121.79	<140.16 ≥140.17	212.68 ± 99.61	<202.28 ≥202.29	171.52 ± 104.47	<145.07 ≥145.08	0 1
Dairy products ^b^	438.48 ± 206.20	≥465.63 <465.62	397.31 ± 208.15	≥377.94 <377.93	470.34 ± 224.86	≥462.50 <462.49	274.05 ± 170.06	≥275.0 <274.9	0 1
Meat (g/day)	41.20 ± 23.95	≥39.29 <39.28	39.30 ± 21.06	≥39.64 <39.63	50.19 ± 35.13	≥45.71 <45.70	48.00 ± 31.93	≥38.57 <38.56	0 1

Values are presented as the mean ± standard deviation. ^a^ Monounsaturated fatty acids/saturated fatty acid; ^b^ dairy products included: milk (mL/day), milkshake (mL/day), yogurt and cheese (g/day).

**Table 2:** General characteristics of the cohort by period.

		Lactation Period	Weaning Period	Transition Diet	Family Diet	
		2–3 Months	6 Months	12 Months	24 Months	36 Months
Subjects (*n*)		97	93	90	76	64
Gender	Male	56 (57.7)	53 (57.0)	51 (56.7)	44 (57.9)	37 (57.8)
	Female	41 (42.3)	40 (43.0)	39 (43.3)	32 (42.1)	27 (42.2)
Weight (kg)		5.82 ± 0.83	7.76 ± 0.84 *	9.97 ± 1.52 *	12.66 ± 1.76 *	15.22 ± 2.52 *
Height (cm)		59.98 ± 2.88	67.73 ± 2.36 *	75.89 ± 3.37 *	88.46 ± 3.84 *	96.27 ± 4.58 *
Lactation	BF	52 (53.6)	39 (41.9)	23 (25.6) *	10 (13.3)	5 (7.8)
	IF	45 (46.4)	54 (58.1)	56 (62.2)	9 (12.0) *	4 (6.3)
	Other	0	0	11 (12.2)	56 (74.7) *	55 (85.9)
Supplementation	No	7 (7.3)	6 (6.5)	10 (11.1)	75 (98.7) *	64 (100)
	Yes	89 (92.7)	87 (93.5)	80 (88.9)	1 (1.3) *	0
Food texture	Mashed food	0	80 (87.9)	8 (8.9) *	0	0
	Semi-solid	0	9 (9.9)	60 (66.7) *	1 (1.3) *	1 (1.6)
	Regular	0	2 (2.2)	22 (24.4) *	75 (98.7) *	63 (98.4)
Special diet	No	0	88 (94.6)	87 (96.7)	73 (96.1)	63 (98.4)
	Yes	0	5 (5.4)	3 (3.3)	3 (3.9)	1 (1.6)
Delivery type	Vaginal	75 (77.3)	71 (76.3)	68 (75.6)	58 (76.3)	48 (75.0)
	C-section	22 (22.7)	22 (23.7)	22 (24.4)	18 (23.7)	16 (25.0)

Data expressed as N (%) or the mean ± standard deviation. BF, breastfeeding; C-section, caesarean section; IF, infant formula. * Differences compared to previous category (*p* < 0.05).

**Table 3:** Energy and macro- and micronutrients intake compared with dietary reference values (DRVs) based on intakes from the EFSA (European Food Safety Authority) in the study’s cohort at 12, 24 and 36 months.

	DRV		12 Months *n*= 90	DRV Compliance 12 Months (%)		24 Months *n* = 76	DRV Compliance 24 Months (%)		36 Months *n* = 64	DRV Compliance 36 Months (%)	
	AI	AR	Median (IR)	>AI	<AR	Median (IR)	>AI	<AR	Median (IR)	>AI	<AR
Energy (kcal/day)	-	-	1019.0 (925.0–1146.1) _a_	-	-	1147.4 (941.9–1363.8) _a_	-	-	1253.0 (1016.8–1470.0) _a_	-	-
Macronutrients				-	-		-	-		-	-
Fat (g/day)	-	-	36.2 (32.5–40.6) _a_	-	-	41.1 (35.1–47.5) _a_	-	-	46.2 (36.0–54.8) _a_	-	-
SFA (g/day)	ALAP	-	10.7 (7.4–12.8) _a_	-	-	15.2 (11.5–18.3) _b_	-	-	15.7 (12.0–19.6) _b_	-	-
MUFA (g/day)	-	-	10.2 (9.0–12.4) _a_	-	-	16.5 (13.7–19.7) _b_	-	-	17.9 (14.5–26.1) _b_	-	-
PUFA (g/day)	-	-	2.8 (2.2–3.4) _a_	-	-	4.3 (3.6–5.1) _a,b_	-	-	5.5 (4.1–6.7) _b_	-	-
Carbohydrate (g/day)	-	-	130.1 (119.5–151.9) _a_	-	-	139.5 (109.0–168.4) _a_	-	-	148.5 (122.5–172.4) _a_	-	-
Dietary fiber (g/day)	10		13.6 (11.4–16.3) _a_	83.3	-	14.3 (11.2–16.8) _a_	84.2	-	15.8 (12.1–18.8) _a_	79.7	-
Protein (g/day)	-	-	36.8 (30.7–43.1) _a_			52.2 (44.1–63.0) _a_			55.7 (47.6–65.9) _a_	-	-
Animal protein (g/day)	-	-	17.0 (12.4–23.6) _a_			32.2 (24.0–39.2) _b_			33.9 (28.4–40.9) _b_	-	-
Vegetal protein (g/day)	-	-	11.8 (9.2–15.3) _a_			17.1 (13.2–22.0) _a,b_			20.8 (15.8–25.3) _b_	-	-
Micronutrients										-	-
Vitamin A (μg RAE/day)	-	205	959.7 (737.7–1282.4) _a_	-	0	564.8 (388.7–841.3) _b_	-	6.6*	564.1 (364.1–861.0) _b_	-	6.3
Thiamin (mg/day)	-	0.072	1.0 (0.8–1.2) _a_	-	0	0.9 (0.8–1.1) _a_	-	0	1.1 (0.8–1.3) _a_	-	0
Riboflavin (mg/day)	-	0.5	1.4 (1.1–1.6) _a_	-	1.1	1.4 (1.1–1.7) _a_	-	2.6	1.5 (1.1–1.8) _a_	-	0
Niacin (mg/day)	-	1.3	9.3 (8.0–12.1) _a_	-	0	10.8 (8.2–13.4) _a_	-	0	11.4 (9.3–14.9) _a_	-	0
Vitamin B-6 (mg/day)	-	0.5	1.4 (1.2–1.7) _a_	-	1.1	1.4 (1.1–1.8) _a_	-	0	1.6 (1.2–1.9) _a_	-	0
Folate (μg DFE/day)	-	90	359.0 (249.2–462.7) _a_	-	1.1	446.2 (342.4–570.3) _a_	-	1.3	566.7 (413.9–676.2) _a_	-	0
Vitamin B-12 (μg/day)	1.5		2.4 (1.8–3.4) _a_	88.9	-	3.0 (2.2–4.2) _a_	92.1	-	3.0 (2.3–3.8) _a_	92.2	-
Vitamin C (mg/day)		15	162.3 (122.0–224.8) _a_	-	0	93.5 (57.7–129.3) _b_	-	1.3	88.5 (59.6–149.0) _b_	-	1.6
Vitamin D (μg/day)	15	-	6.0 (3.8–7.3) _a_	0	-	2.8 (0.9–4.1) _b_	1.3	-	2.3 (1.1–4.0) _b_	0	-
Vitamin E (mg/day)	6 ^$^	-	8.7 (6.7–11.0) _a_	80.0	-	5.1 (4.0–6.7) _b_	38.2 *	-	5.7 (4.5–7.5) _b_	12.5 *	-
Vitamin K (μg/day)	12	-	45.0 (27.4–82.1) _a_	94.4	-	31.7 (19.9–75.4) _a_	94.7	-	38.8 (21.1–116.5) _a_	90.6	-
Choline (mg/day)	140	-	112.4 (88.2–155.8) _a_	27.8	-	234.8 (184.4–286.5) _b_	88.2 *	-	266.3 (187.0–299.4) _b_	89.1	-
Calcium (mg/day)	-	390	643.3 (525.3–736.1) _a_	-	8.9	730.0 (577.8–975.8) _a_	-	7.9	650.1 (527.4–889.1) _a_	-	7.8
Copper (mg/day)	0.7 ^‡^	-	0.6 (0.4–0.7) _a_	27.8	-	0.9 (0.7–1.0) _a_	73.7 *	-	1.0 (0.7–1.2) _a_	48.4 *	-
Phosphorus (mg/day)	250	-	631.0 (551.8–782.9) _a_	100	-	930.0 (777.1–1121.5) _a_	100	-	948.9 (774.4–1115.4) _a_	100	-
Potassium (mg/day)	800	-	2070.7 (1712.9–2542.4) _a_	98.9	-	2365.8 (1836.7–2657.4) _a_	98.7	-	2506.2 (1941.0–2739.1) _a_	100	-
Iron (mg/day)	-	5	9.4 (7.6–11.3) _a_	-	2.2	7.6 (6.3–8.8) _a_	-	13.2 *	8.3 (6.6–9.9) _a,b_	-	6.4
Magnesium (mg/day)	170 ^&^	-	147.7 (123.0–186.9) _a_	35.6	-	194.8 (155.8–232.1) _a_	65.8*	-	203.7 (166.6–240.1) _a_	28.1 *	-
Selenium (μg/day)	15	-	30.9 (21.5–46.1) _a_	95.6	-	66.4 (49.5–79.7) _b_	100	-	68.5 (50.1–81.5) _b_	100	-
Zinc (mg/day)	-	3.6	5.8 (4.5–6.8) _a_	-	10.0	6.4 (5.3–7.9) _a_	-	3.9	6.6 (5.6–7.8) _a_	-	4.7

Values are presented as the median (interquartile range). ^$^ 9 mg/day at 3 years; ^&^ 230 mg/day at 3 years; ^‡^ 1 mg/day at 3 years. AI, adequate intake; ALAP, as low as possible; AR, average requirement; MUFAs, monounsaturated fatty acids; PUFAs, polyunsaturated fatty acids; SFAs, saturated fatty acids. Different letters indicate significant differences between infants’ ages from the Kruskal–Wallis test (*p* < 0.05). * Differences compared to the previous category from a chi-square test (*p* < 0.05).

**Table 4:** Linear association between diet at 6 months and the Mediterranean Diet Score (MDS) at 24 and 36 months.

			MDS	
	Predictors	*R* ^2^	*β*	*p*
MDS at 24 months				
Model	Energy	0.069	−0.286	0.013
MDS at 36 months				
Model	Vegetables	0.105	0.365	0.006
	Fruits	0.105	−0.265	0.044

Result from linear stepwise regression analyses including energy, fruit, vegetables, fats, tubers, infant products and human breast milk at 6 months as potential predictors, and the Mediterranean Diet Score as the dependent variable. *β,* standardized regression coefficient; *R*^2^, adjusted coefficient of multiple determination; *p, p*-value.

## Data Availability

Not applicable.

## Supplementary Materials

The following supporting information can be downloaded at: https://www.mdpi.com/article/10.3390/nu14081664/s1. Table S1: Evolution of the intake of the major food groups with follow up; Figure S1: Adherence to daily food recommendations in the sample with follow up [25].
