# Supplementary material for: Early Life Nutrition and the Role of Complementary Feeding on Later Adherence to the Mediterranean Diet in Children up to 3 Years of Age
Source: Nutrients. 2022 Apr 16;14(8):1664. doi: 10.3390/nu14081664 (PMC9027550; doi:10.3390/nu14081664)
Supplement: Supplementary file 1 [file nutrients-14-01664-s001.zip › nutrients-1658500-supplementary.pdf]

Supplementary Table S1. Evolution in the intake of the major food groups along the follow-up.

	Lactation period	Weaning period	Transition diet	Family diet	
	2-3 months (*n*=97)	6 months (*n*=93)	12 months (*n*=90)	24 months (*n*=76)	36 months (*n*=64)
**Fats**[a]	0	0 (0-10.0)*	10 (10-10)*	10 (10-10)	10 (10-20)
**Vegetables (g/day)**	0	0 (0-66.3)*	149.2 (96.4-205.0)*	85.7 (38.5-128.6)	72.0 (30.5-155.0)
**Potatoes and tubers (g/day)**	0	0 (0-28.1)*	44.6 (25.0-75.0)*	33.6 (20.6-50.3)	38.9 (21.3-53.6)
**Cereals and cereals products (g/day)**	0	0	30.7 (15.0-54.3)*	67.7 (41.0-105.4)	83.9 (58.9-115.7)
**Fruits (g/day)**	0	52.8 (0-135.8)*	158.6 (109.9-205.6)*	157.6 (96.4-262.6)	173.7 (114-9-277.0)
**Legumes (g/day)**	0	0	15.0 (5.7-25.7)*	27.8 (18.6-41.6)	34.3 (19.3-52.9)
**Meat and meat products (g/day)**	0	0	25.7 (17.0-37.1)*	39.6 (25.7-56.4)	43.6 (26.4-60.0)
**Fish (g/day)**	0	0	13.6 (8.6-40.7)*	27.1 (13.6-51.4)	27.1 (25.7-51.4)
**Eggs (g/day)**	0	0	9.1 (9.1-18.3)*	18.3 (18.3-27.4)	18.3 (18.3-27.4)
**Milk and dairy products**[b]	0	0	125.0 (51.3-150.0)*	430.36 (264.3-559.5)*	360.7 (218.8-563.3)
**Human breast milk (ml/day)**	180 (0-780)	0 (0-680)	0*	0	0
**Processed infant products**	600 (0-840)	613.5 (118.5-853.5)	383.5 (36.0-522.5)	9 (0-27.0)*	0 (0-11.25)
Infant formulas (ml/day)	600 (0-840)	600.0 (100.0-840.0)	330.0 (0-500.0)*	0*	0
Infant cereals (g/day)	0	9 (0-18.0)*	15.8 (4.5-27.0)*	0 (0-18.0)*	0*
Infant purees (g/day)	0	0*	0	0*	0
**Sweets and desserts (g/day)**	0	0	6.0 (3-14.3)*	12 (6.0-28.7)	18.0 (6.0-37.0)
**Sweetened beverages (ml/day)**	0	0*	0	0	0 (0-33.29)

Data expressed as median (interquartile range). [a] Fats included: oils (ml/day) and butter (g/day). [b] Milk and dairy products included: milk (ml/day), milkshake (ml/day) yogurt and cheese (g/day). *p value < 0.05 from Kruskall Wallis test compared to the previous category.

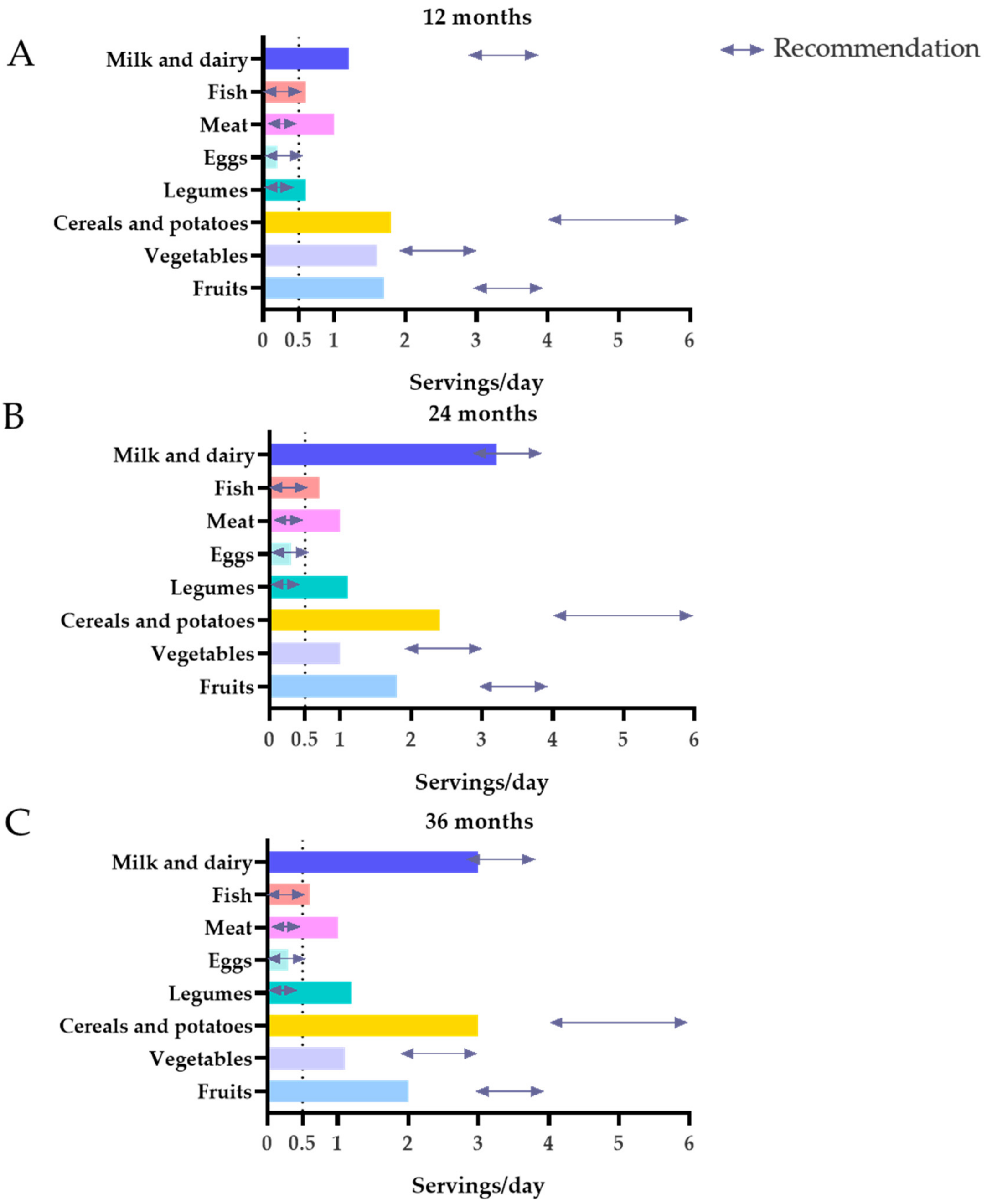

Supplementary Figure S1. Adherence to daily food recommendations in the sample along the follow-up. General portion size: Milk and dairy (milk 125 ml, yogurt 125 g and fresh cheese 40 g), legumes 30 g, cereals and potatoes (potatoes 100 g, rice and pasta 75 g and bread 25 g), vegetables 100 g, fruits 100 g, eggs (1 unit). (A) Specific portion size for 12 month: fish 40 g, meat 30 g, (B) Specific portion size for 24 month: fish 50 g, meat 40 g and (C) Specific portion size for 36 month: fish 60 g, meat 50 g [25].
